# An Interdisciplinary Approach to the Introduction of Point-of-Care Ultrasound in an Urban Academic Primary Care Center

**DOI:** 10.7759/cureus.36329

**Published:** 2023-03-18

**Authors:** Sarah E Frasure, Rachel Treat, Jordan Dow, Elizabeth Dearing, Luis Dominguez, Aaran Drake, Kathleen Y Ogle, Maria Portela, Joel Willis, Keith S Boniface

**Affiliations:** 1 Emergency Medicine, George Washington University School of Medicine and Health Sciences, Washington D. C., USA; 2 Emergency Department, George Washington University School of Medicine and Health Sciences, Washington, D. C., USA; 3 Emergency Medicine, George Washington University School of Medicine and Health Sciences, Washington, D. C., USA; 4 Internal Medicine/Primary care, George Washington University School of Medicine and Health Sciences, Washington, D. C., USA; 5 Family Medicine, George Washington University School of Medicine and Health Sciences, Washington, D. C., USA

**Keywords:** faculty development, urgent health care, primary health care, point-of-care ultrasonography, medical education

## Abstract

Introduction

Limited guidance exists for primary and urgent care ultrasound applications. This study sought to identify the most useful applications for providers in these clinical settings, to create and implement a structured interdisciplinary point-of-care ultrasound (POCUS) curriculum, and to assess the effectiveness of the course.

Methods

This prospective cohort study took place at an urban academic medical center. After a needs-based assessment of ultrasound applications in primary and urgent care, the Emergency Medicine ultrasound faculty and fellows were paired with a primary or urgent care provider (N = 6). The pairings met for scanning sessions in the emergency department to practice image acquisition, documentation, and incorporation of ultrasound into the workflow. Participants were given POCUS pre-work to review before each session. The final bedside session included a formal Objective Standard Clinical Examination (OSCE) to assess learner proficiency to be cleared for independent imaging. The program was assessed using pre- and post-training surveys.

Results

The survey results demonstrated that renal, gallbladder, and soft tissue scans were the most interesting and useful to primary and urgent care providers after completion of the training course.

Conclusion

The course was effective, and efficient, simple, high-yield POCUS applications should be included in future programs and organizational guidelines for primary and urgent care POCUS education.

## Introduction

Point of care ultrasound (POCUS) was first popularized in the late 20th century with the emergency physician’s focused assessment with sonography in trauma (FAST) scan for hemoperitoneum or pericardial effusion [1]. Clinicians in a variety of specialties have since taken an interest in expanding their clinical repertoire with POCUS, which is becoming increasingly feasible with the advent of ultraportable handheld ultrasound devices [2,3]. POCUS has been shown to shorten the length of hospital stay, lower medical costs, provide greater patient satisfaction, avoid unnecessary irradiation, safely guide procedures, and offer faster, more accurate diagnoses [2,4,5,6,7]. Andersen et al. report that POCUS cardiac and abdominal exams performed by emergency medicine residents using handheld systems resulted in a correction, validation, or supplementation to diagnoses in at least one of every three medical admissions [8]. Another randomized clinical trial demonstrated that a POCUS protocol for emergent patients significantly shortened the time to lifesaving treatment and also increased diagnostic accuracy while lowering mortality rates [9]. POCUS significantly lowers costs compared to ultrasound referrals [5,10]. While diagnoses are based on a patient's history, physical exam, and labs, the ready availability and diagnostic accuracy of POCUS means that clinicians will increasingly look within patients for definitive and expeditious answers [4,11].

The Council of Emergency Medicine Residency Directors has recognized the need for standardization of training for POCUS in primary care. They propose a graded level of progression for the evaluation of residents and a list of suggested basic and advanced ultrasound exams in primary care [11]. More specifically, they recommend four weeks of focused work on technique during the first year of residency and then an advanced course for senior residents later on [12]. There is no universally standardized list of topics and skills that all POCUS learners should master for a given specialty, although some preliminary content recommendations do exist [2,13-16]. In addition to a lack of training guidelines, barriers to implementing POCUS in a primary care setting include a lack of instructors, equipment, and practitioner confidence in independently interpreting images [17]. A recent survey of family medicine residents and recent graduates demonstrates strong support for the development of POCUS curricula, but as of 2021, less than 10% of primary care providers report using POCUS in the clinic [9,18]. Several previous studies have demonstrated that general practitioners are capable of scanning patients with accuracy and efficiency with as little as three didactic and five hands-on hours of POCUS training [10,12]. Family medicine residents express more confidence and likelihood of using POCUS in their future practice if they are allowed hands-on practice under expert supervision [19,20]. The importance of POCUS in primary care is evidenced by a quadrupling in the number of publications on ultrasound education since 1990 and the American Academy of Family Physicians’ endorsement of a POCUS curriculum for resident training [1,2]. With a greater focus on standardized primary care POCUS guidelines, patients will increasingly benefit from time-saving, cost-saving, and more accurate diagnoses much like those in emergency departments already do. 

Efforts to incorporate and standardize ultrasound education within primary and urgent care have steadily increased. Solitary workshops, instruction at organizational conferences, online content, and longitudinal training programs are springing up around the nation. With so many POCUS topics and pedagogies, how can we tease out the POCUS applications that are truly most useful in primary and urgent care? A curriculum that prioritizes simple, efficient, and high-yield diagnostic applications is the most practical approach. 

As a branch of the George Washington University Emergency Department, the Immediate and Primary Care clinics (IPC) are three urban academic outpatient medical clinics providing both primary and urgent care services. At the outset of the study, the majority of providers at the IPC were ultrasound-naive practitioners, and the clinic was not equipped with ultrasound technology. The objective of this study was to identify the most useful ultrasound applications for providers in primary and urgent care clinics, to create and implement an interdisciplinary POCUS curriculum, and to assess the effectiveness of the course by surveying the participating IPC providers before and after the training. The study aims to clarify the POCUS applications that should be included in future training programs and organizational guidelines for primary and urgent care. 

## Materials and methods

This prospective cohort study took place both at the emergency department and outpatient clinics for urgent and primary care services at an urban academic medical center. The training program sought to increase the efficiency and diagnostic capacity of participants by providing them with the skills and equipment necessary to implement POCUS in clinics. Emergency medicine (EM) and ultrasound (US) faculty and fellows in an Emergency Ultrasound Fellowship Accreditation Council (EUFAC) accredited fellowship program were paired with a primary or urgent care provider in an eight-week longitudinal training course. The demographics of the six participating primary and urgent care providers are detailed in Table 1. The study was reviewed by the institutional review board (IRB) and determined to be exempt (IRB number NCR213518).

**Table 1:** Demographics of the primary care providers and outline of the POCUS training curriculum *Only 4 of 6 respondents answered this question **Learners were also provided the option to join ED providers on their clinical shifts for additional practice during the 8-week training period Abbreviations: Immediate and Primary Care (IPC), Point of care ultrasound (POCUS), Emergency Department (ED), Objective Standard Clinical Examination (OSCE) This table demonstrates the demographics of the primary care physicians who participated in the training course and outlines the POCUS training curriculum.

Demographics of IPC providers who participated in the training course (n = 6)		
Age and Gender	30 - 40 years	66.7%
	40 - 50 years	33.3%
	Female	50%
Training Background and License	Received prior ultrasound training	33.3%
	Nurse practitioners *	50%
	Physician assistants *	25%
	Attending physicians *	25%
Years in Practice	Less than 5 years in practice	50%
	5-10 years in practice	33.3%
	More than 10 years in practice	16.7%
POCUS Training Curriculum		
Session 1 - Basic Techniques (3 hours)	Practice core imaging techniques on a standardized patient supplemented with images/videos of ultrasound pathology.	
Session 2, 3, 4 – ED and IPC Hands-On Image Acquisition (12 hours) **	Learner scans patients in the ED, practicing quality image acquisition, documentation, and incorporation of US into a workflow.	
Session 5 - OSCE of POCUS competence (3 hours)	Learners will demonstrate proficiency in all of the US techniques covered in the bedside rotation in order to be cleared for independent imaging. OSCE Evaluation criteria: Selected appropriate probe. Performed appropriate exam type. Attained appropriate anatomical view with full visualization of pertinent structures in all necessary planes. Optimized depth, gain, focus, and color Doppler as needed Identified presence or absence of appropriate pathology. Measured pertinent structures. Appropriately labeled different views. Quality of images acquired. Appropriate probe maneuvers. Accuracy of interpretation of images. Properly documented scan with video and/or images. Applied clinical knowledge. Performance met criteria for overall competence.	

The EM principal investigator met with the Chief of Family Medicine over the course of several months to compile a list of ultrasound applications that were expected to be most useful to providers in urgent care and primary care settings based on literature review, EM physician experience with ultrasound, and a needs assessment of the clinics. Each clinical site involved in the training course was provided with a Butterfly ultrasound transducer connected to an iPad. The participants (3 physicians, 2 physician assistants, and 1 nurse practitioner) and EM ultrasound faculty pairings met for five separate three-hour sessions in the emergency department (15 hours total) to practice identifying and documenting anatomy and pathology for the different applications (Table 1). The documentation was reviewed by the instructors as part of a quality assurance (QA) program. Participants were given session pre-work which included reading materials, videos detailing proper technique, and example images. The final bedside session included a formal Objective Standard Clinical Examination (OSCE) which occurred in either the emergency department or the simulation center using a standardized patient, depending on learner preference. The OSCE specifically tested participants in their ability to perform the FAST, soft tissue/MSK, renal, aorta, gallbladder, and transabdominal obstetric POCUS examinations. During this final session, the instructor assessed the learner’s skillset and determined if further practice was required before independent scanning. Participants were evaluated for competence based on applied clinical knowledge, appropriate anatomical views, image quality, proper measurements and probe maneuvers, accurate diagnostic interpretation, and correct documentation (Table 1). Survey responses were analyzed using summary statistics such as frequencies and percentages for dichotomous and categorical outcomes. 

Providers completed a voluntary and anonymous pre-training survey to assess their interest in, familiarity with, and incorporation of POCUS into the primary and urgent care settings. An anonymous survey was also administered after the training program to assess their comfort levels in the applications they had learned, and their experience with the curriculum. Every three months for one year following the course completion, each provider was also asked to fill out an anonymous survey in order to learn which POCUS skills they found most and least useful in the clinic and what additional POCUS topics they would be interested in learning about for future sessions. 

## Results

**Figure 1:**
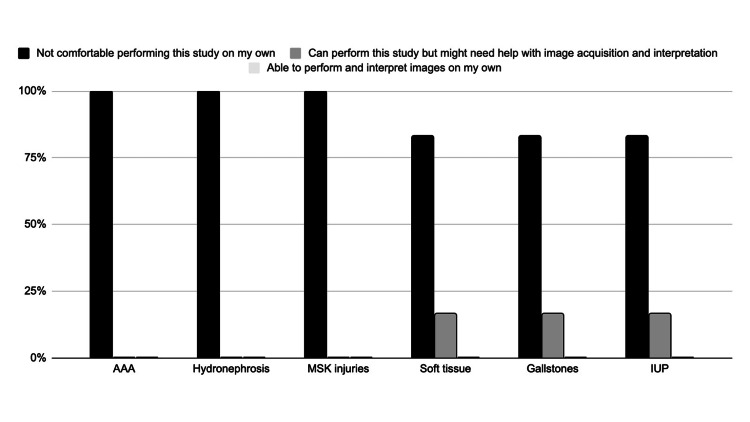
Responses for pre-training provider familiarity with selected POCUS applications (N = 6) POCUS application: AAA = Abdominal aortic aneurysm, MSK = Musculoskeletal, IUP = intrauterine pregnancy

A total of six providers from the urgent and primary care clinics participated. Each participant voluntarily completed an anonymous electronic survey prior to and several times after the POCUS training course. The pre-training survey determined the providers’ familiarity with various POCUS applications and their confidence level in performing each scan (Figure 1). The response “able to perform this study on my own” was available for each POCUS application, but was not selected by any respondent.

**Figure 2:**
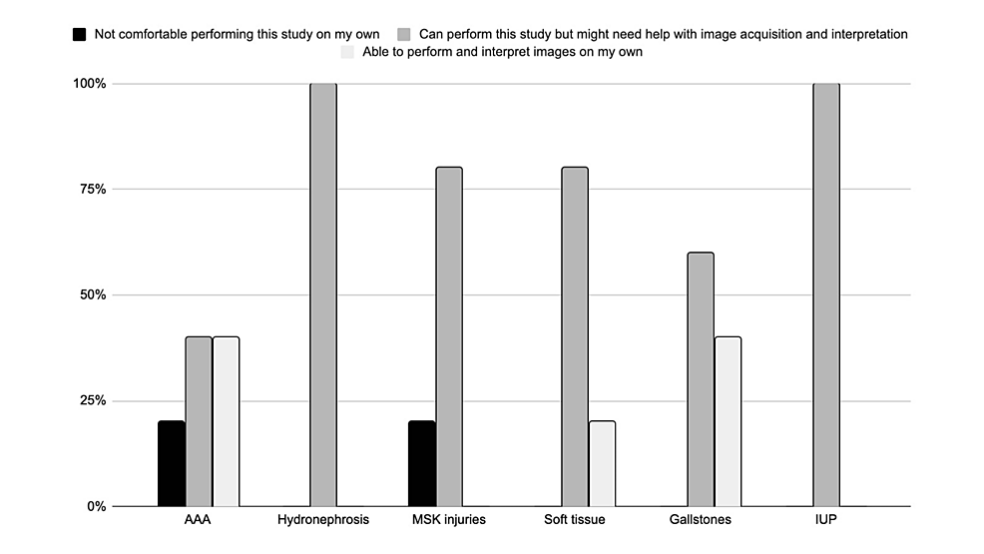
Responses for post-training provider familiarity with selected POCUS applications (N = 5) POCUS application: AAA = Abdominal aortic aneurysm, MSK = Musculoskeletal, IUP = Intrauterine pregnancy

In contrast, Figure 2 shows the responses for post-trainer provider familiarity with the same ultrasound applications after four three-hour scanning sessions with an EM US-trained faculty member or fellow. An OSCE was performed to evaluate the providers’ ability to perform the FAST, soft tissue/MSK, renal, aorta, gallbladder, and transabdominal obstetric POCUS examinations (see Table 1). 

**Figure 3:**
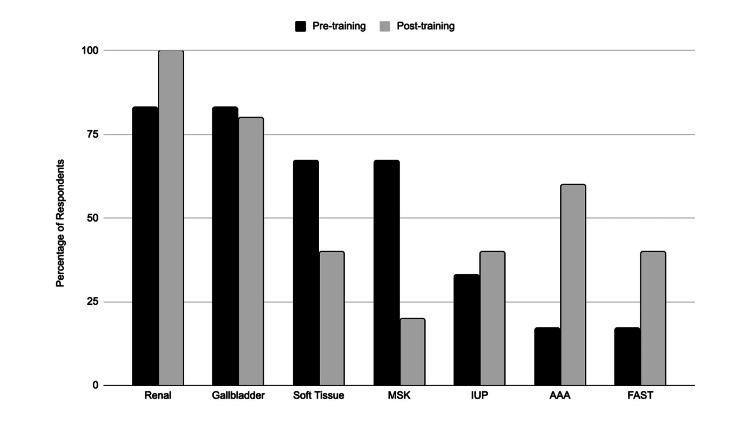
Responses for pre-training (N=6) and post-training (N=5) provider interest in POCUS applications POCUS application: MSK = Musculoskeletal, IUP = Intrauterine pregnancy, AAA = Abdominal aortic aneurysm, FAST = Focused assessment with sonography for trauma

Finally, a pre- and post-training survey determined which POCUS applications the providers were most interested in, with renal and gallbladder applications taking the lead (Figure 3). 

**Figure 4:**
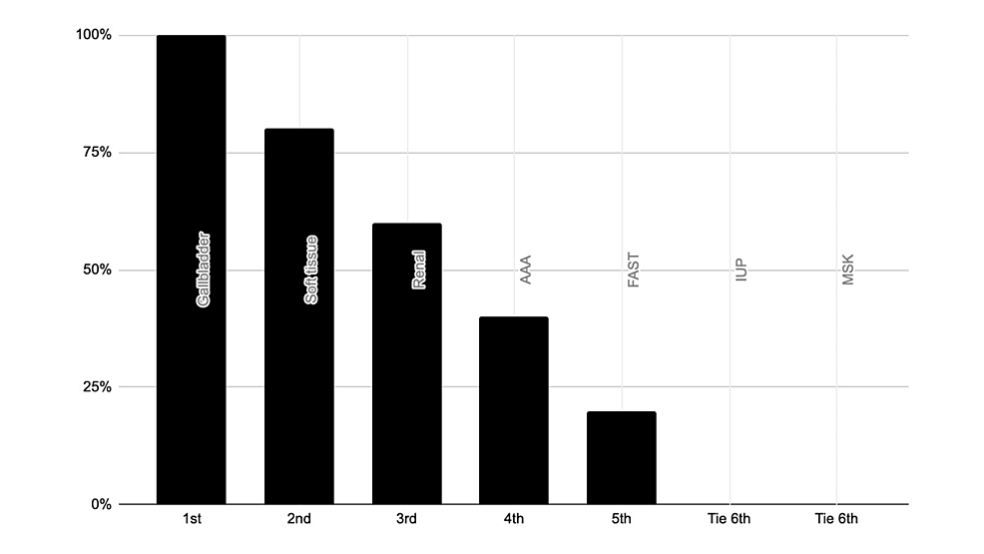
Responses for post-training provider expected frequency of use for POCUS applications (N = 5) POCUS application: MSK = Musculoskeletal, IUP = Intrauterine pregnancy, AAA = Abdominal aortic aneurysm, FAST = Focused assessment with sonography for trauma

One provider was lost to follow-up, resulting in a response rate of 83%. Respondents were also surveyed on which POCUS applications they thought they might use most frequently in the clinic (Figure 4). When separately asked which applications they would use the least, the respondents answered FAST, intra-uterine pregnancy (IUP), and abdominal aortic aneurysm (AAA). 

All the respondents reported “Agree” or “Strongly Agree” on a Likert scale that they felt the hands-on sessions were relevant to their needs, that they felt comfortable using ultrasound in the clinic after training, and that they would be interested in future POCUS training sessions for advanced applications such as cardiac POCUS for ejection fraction, pericardial effusion, or right ventricular strain (100% of respondents), lung POCUS for pneumonia (40% of respondents), gastrointestinal POCUS for small bowel obstruction or appendicitis (60% of respondents), and ocular POCUS for retinal detachment/hemorrhage and vitreous detachment/hemorrhage (60% of respondents). 

**Figure 5:**
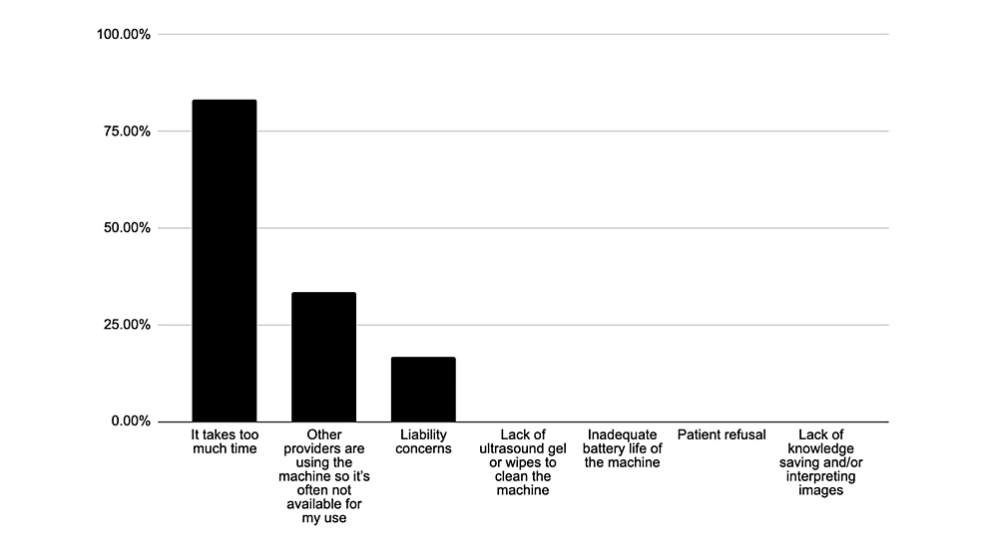
Responses for perceived barriers to POCUS use in the clinic (N=6)

The surveys were entirely voluntary and one provider was lost to follow-up at the three-month follow-up. In the three-month follow-up surveys, 71.43% reported understanding how to use the Butterfly ultrasound machine to save images/video clips, and complete a worksheet for the ultrasound faculty members to review. Approximately, 83.3% of providers responded that the barrier to using POCUS in the clinic was that “it takes too much time.” Another 33.3% of providers reported that a barrier to use is “other providers are using the machine so it’s often not available for my use.” Lastly, 16.6% reported that liability concerns were a barrier to POCUS use in the clinic (Figure 5).

**Figure 6:**
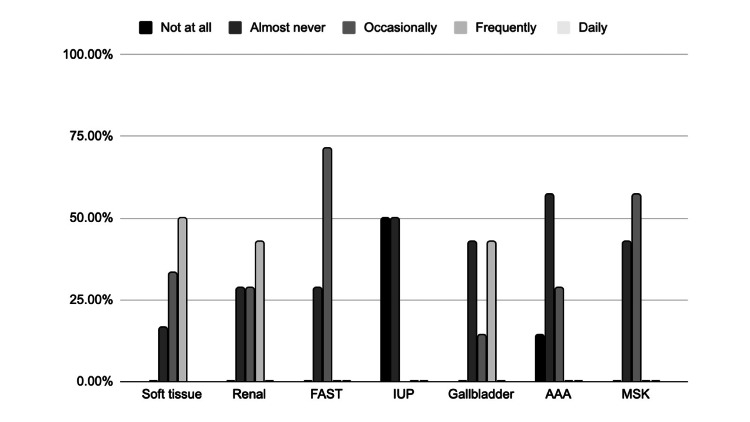
Reported Use of POCUS Applications in Clinic (N = 7) POCUS application: MSK = Musculoskeletal, IUP = Intrauterine pregnancy, AAA = Abdominal aortic aneurysm, FAST = Focused assessment with sonography for trauma

All barriers aside, 100% of respondents state that they would recommend the POCUS rotation to other providers. In the 3-month follow-up survey, providers were also asked which POCUS applications they were currently using in the clinic and with what frequency (Figure 6). 

## Discussion

This study sought to design and implement an interdisciplinary POCUS training course and thereby, through survey data collection, elucidate the POCUS applications that are most useful for providers in primary and urgent care settings. Overall, the participating providers felt the asynchronous materials were useful, the hands-on training sessions were effective, the course was relevant to their needs, and they would be interested in future sessions. The providers progressed from very limited knowledge and skills regarding the selected applications (Figure 1) to a much-improved level of autonomy after completing the training (Figure 2). The POCUS applications that providers perceived to be most interesting and most useful in their clinical setting also shifted after their completion of the course. Before training, providers were most interested in learning to scan for gallstones or hydronephrosis (Figure 3). After training, providers still thought they would often rely on renal and gallbladder imaging in the clinic setting, but also believed they would use soft tissue POCUS frequently (Figure 4). Furthermore, soft tissue, renal, and gallbladder POCUS applications were reported as “frequently” used above all other POCUS applications in the 3-month follow-up survey (Figure 6). With this knowledge, future POCUS education programs in primary care should prioritize the highest yield and most realistic applications. 

The results of this study allow for important distinctions to be drawn between POCUS applications for the ED or a primary/urgent care setting. The IUP and FAST applications are a staple to POCUS training in the ED, allowing students to practice visualizing a variety of structures within one exam, and have been included as recommended topics for budding primary care POCUS programs; however, the IUP and FAST POCUS applications were consistently ranked among the lowest in interest and expected frequency of use in this study (Figures 3 and 4) [2,12,20]. One explanation could be that the FAST scan is potentially most useful to primary and urgent care providers in ruling out ascites, ruptured ectopic pregnancy, or free fluid following a motor vehicle collision [21]. That being said, such high-acuity patients are generally uncommon in outpatient clinics. All of these factors collectively support FAST and IUP scans as less practical topics for primary and urgent care POCUS curricula. 

The post-training survey results also demonstrate that providers felt least interested in the musculoskeletal (MSK) applications (Figure 3) and did not expect to use the AAA or MSK scans with frequency (Figure 4). This conclusion was further affirmed by the low clinical use of the applications reported in the 3-month follow-up survey (Figure 6). This is likely due to the fact that providers are scheduled for approximately 15 minutes per patient and the AAA and MSK scans are more time-consuming and technically challenging. Additionally, “it takes too much time” was selected as the leading barrier to the providers using any POCUS applications (Figure 5). The AAA scan is often complicated by patient body mass and bowel gas, and takes over five minutes on average [22]. In addition to time constraints, providers may also be hesitant to use the abbreviated POCUS application in lieu of a comprehensive scan by radiology due to perceived medicolegal risks. Liability concerns were the third most common barrier to POCUS use selected by respondents (Figure 5). Lastly, the MSK exam for tendon injury can be technically challenging depending upon the tendon involved, is variable in accuracy, and requires more extensive training for proficiency, all of which makes it less useful to primary and urgent care clinicians [23]. In summary, faster, simpler, higher-impact POCUS applications should be prioritized in primary and urgent care training programs. Perhaps medical assistants could also prepare the ultrasound machine and enter relevant patient data into the machine to help the provider save set-up time. 

In accordance with our findings, another survey conducted by Weemer et al. in a family medicine program reports that scanning a patient with biliary colic was perceived as a highly useful family medicine POCUS application by residents and graduates of the program [10]. AAA and the FAST POCUS applications were also ranked low in perceived usefulness.

Multidisciplinary teams are gathering to identify POCUS skills that should be prioritized in curricula. One notable contribution was the POCUS skills checklist assembled in 2022 by a team of EM, critical care, and hospital medicine providers [14]. Consensus voting was used to assemble the selected multisystem POCUS applications for heart, lung, abdomen, and procedural guidance. This method was an excellent example of multidisciplinary collaboration but was not directly relevant to POCUS use in primary or urgent care settings. A statement from the Council of Emergency Medicine residency directors also proposes a graded level of progression for primary care resident POCUS training based on pre-existing ED resident POCUS training [11]. The council lists the AAA, IUP, and FAST scans as having priority based on the ED resident curriculum. This recommendation for time-consuming, high-acuity, challenging scans is not the most practical or feasible recommendation for POCUS in primary care. 

Based on the findings of this longitudinal survey, future primary and urgent care POCUS curricula should prioritize efficient diagnostic applications. Clinicians have many demands placed on them to fit within a short window of time, and so it makes sense that easier POCUS applications that are high yield and can be performed quickly (i.e. looking for gallstones, abscesses, foreign bodies, or hydronephrosis) would be the most powerful use of ultrasound in their workplace. 

One limitation of the study is the small sample size of participants. We also lost one provider to followup at the three-month post-training survey. As this was an interdisciplinary effort in an urban academic medical center, another consideration would be that the results of this study may not be generalizable to all POCUS education programs. An additional barrier in this study may have been a lack of equipment. Although each clinic that was involved was supplied with a Butterfly ultrasound machine, the providers reported that the machine was often in use and unavailable to them (Figure 5). In the future, the training program and surveys could be conducted on a larger scale at other institutions in order to acquire data with more generalizable trends. 

A future supplementation to the POCUS curriculum described in this study would be to offer participants additional follow-up sessions once a month with the ultrasound faculty instructors. These follow-up sessions could also be used to review applications and how to use the ultrasound machine (and even troubleshoot technical difficulties), and examine previously collected scans. This form of longitudinal training has been supported by several other studies. Flick et al. recommend that residents have at least 10 hours of training in their first year with electives to follow in their second and third years [12]. Barron also describes an extended curriculum for ultrasound fellows that includes longitudinal faculty supervision with feedback on imaging, didactic sessions, and scanning time [2]. If programs do not have the bandwidth of ultrasound-trained faculty members to conduct such thorough quality assurance measures, sonography student-coaches could be relied upon to fill in as instructors [24]. 

Single protocol or procedure days for the more time-consuming, complicated POCUS applications could also be implemented in the future. For example, primary or urgent care providers could set aside one day each month in order to perform AAA screenings, MSK injections, or paracentesis, respectively. The provider could schedule ahead all the patients they would like to do certain ultrasound studies or procedures on and have the ultrasound faculty present for assistance if needed. This would streamline patient care and allow providers to implement useful POCUS applications that may not be feasible otherwise. 

## Conclusions

In summary, this study successfully implemented an interdisciplinary POCUS curriculum in an urban academic primary care center. The findings of the study support the idea that future POCUS education in the primary and urgent care settings should focus on applications that are efficient, simple, and high yield, such as renal, gallbladder and soft tissue scans, for a population of providers who are often pressed for time.
